# The biogenesis and biological roles of migrasomes in human diseases

**DOI:** 10.1038/s41420-025-02569-8

**Published:** 2025-07-01

**Authors:** Yifan Zhang, Wei Chen, Jiageng Zhu, Luwei Xu

**Affiliations:** Department of Urology, Nanjing First Hospital, Nanjing Medical University, Nanjing, Jiangsu People’s Republic of China

**Keywords:** Cell migration, Pathogenesis

## Abstract

Migrasomes are recently identified extracellular vesicles that are specifically generated by migrating cells. These pomegranate-like, membrane-bound organelles are released at the trailing edge during cell migration and play crucial roles in cell-to-cell communication, intercellular signaling, and tissue remodeling. Migrasomes selectively package various molecular components, including proteins, lipids, and RNA, facilitating a unique form of cellular communication known as migracytosis. They are involved in numerous physiological and pathological processes, including immune responses, cancer metastasis, tissue repair, and embryonic development. In this review, we provide an in-depth analysis of the biogenesis, structural features, and molecular composition of migrasomes. We further explore the emerging roles of migrasomes in disease pathogenesis, particularly their potential in cancer, neurodegenerative diseases, and immune modulation. Overall, this review aims to offer comprehensive insights into the latest research on migrasomes, while addressing the challenges in their study and potential avenues for future clinical implementation.

## Facts

- Migrasomes are unique extracellular vesicles: They are specifically generated by migrating cells and are released at the trailing edge during cell migration. Their distinctive pomegranate-like structure is central to their function.
- Migrasomes play a significant role in cellular communication: These vesicles facilitate migracytosis, a unique form of cellular communication that involves the packaging and transfer of proteins, lipids, and RNA.
- Migrasomes are implicated in multiple physiological and pathological processes: They are involved in immune responses, cancer metastasis, tissue repair, and embryonic development.
- The molecular composition of migrasomes is diverse: Migrasomes selectively package a variety of molecular components, suggesting their complex and highly regulated biogenesis and function.
- Migrasomes may hold therapeutic potential: Emerging evidence suggests that migrasomes could be important in the pathogenesis of diseases such as cancer and neurodegenerative disorders, highlighting their potential for future clinical applications.

## Open Questions

- How do migrasomes selectively package molecular components?: The mechanisms that govern the selective incorporation of proteins, lipids, and RNA into migrasomes remain unclear and require further investigation.
- What are the precise signaling pathways involved in migrasome biogenesis?: While migrasomes are known to be associated with cell migration, the detailed molecular mechanisms driving their formation and release need to be elucidated.
- What is the role of migrasomes in tissue-specific contexts?: The involvement of migrasomes in different tissues or organs during development, injury, or disease requires more research to understand their specific roles and functions.
- Can migrasomes be effectively targeted for therapeutic purposes?: Given their potential in disease modulation, it is still uncertain whether migrasomes can be targeted effectively for therapeutic purposes, particularly in diseases like cancer and neurodegenerative disorders.
- What is the long-term impact of migrasomes on intercellular communication in the context of chronic diseases?: Investigating how migrasomes influence cell-to-cell communication over extended periods, especially in chronic conditions, could offer insights into their long-term biological effects and therapeutic potential.

## Introduction

**Fig. 1:**
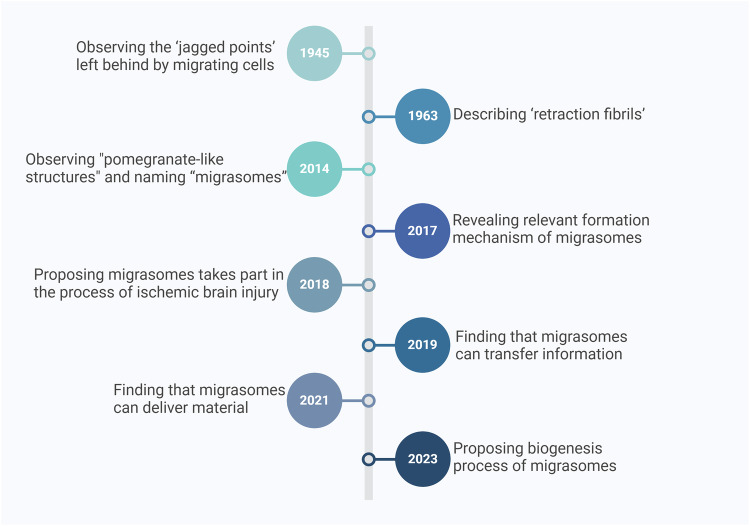
Chronology of key milestones in migrasome research. Created with BioRender.com.

Cell migration is a vital cellular process underlying numerous essential physiological functions, including embryogenesis, tissue development, wound healing, immune responses, and cancer metastasis [1–3]. During cell migration, cells dynamically interact with their environment, forming specialized structures at the leading and trailing edges of the migrating cell [4]. One such structure, the retraction fiber (RF), consists of long, cylindrical membrane tethers that trail behind migrating cells. These fibers were first observed by Porter and colleagues in 1945, but the limitations of early microscopy techniques left their precise biological significance unclear for decades. In 1963, Taylor and Robbins provided further clarity by observing these fibers under high-resolution electron microscopy, coining the term “retraction fibrils” [5]. Despite being recognized in various cell types, the functional role of RFs was largely overlooked until the discovery of migrasomes in 2015 [6] (Fig. 1).

Migrasomes are novel organelles specifically generated during cell migration, appearing at the branches or tips of RFs [7]. These organelles, ranging from 0.5 to 3 μm in diameter, possess a distinct pomegranate-like structure and are released when the retraction fibers are disrupted, marking them as extracellular vesicles (EVs) after detachment from the migrating cell [8, 9] (Table 1). Although first identified in rat kidney cells, migrasomes have since been observed in various cell types, including immune cells, fibroblasts, and tumor cells [10]. Since their discovery, migrasomes have garnered significant attention due to their unique biogenesis, structural characteristics, and biological roles. The formation of migrasomes is intricately tied to cell migration. Initially, small integrin-positive sites accumulate at the base of retraction fibers, providing the foundation for migrasome formation. Over time, these sites evolve into mature migrasomes through a complex process regulated by tetraspanins, small GTPases such as Rab35, and lipid molecules like phosphatidylinositol (4,5)-bisphosphate (PIP2) [11]. Unlike other EVs, such as exosomes, which form via inward budding of multivesicular bodies, migrasomes are directly released through the rupture of RFs, conferring a distinct mechanism of release [7]. This process allows migrasomes to carry cellular cargo, including proteins, lipids, and RNA, which can be transferred to neighboring cells to modulate cellular communication and influence various physiological processes. Moreover, migrasomes play critical roles in a range of biological functions. In developmental biology, they participate in organ morphogenesis, angiogenesis, and immune cell migration [12, 13]. In pathological contexts, migrasomes have been implicated in cancer metastasis, where they may carry tumor-associated markers like PD-L1, facilitating immune evasion and tumor progression [14]. Additionally, their involvement in tissue repair, inflammation, and response to environmental stress is emerging as a critical area of research [15]. The ability of migrasomes to influence these processes positions them as key players in both normal physiology and disease.

**Table 1:** The differences between migrasomes and exosomes.

Property	Migrasome	Exosome	References
Diameter	500–3000 nm	30–150 nm	[6, 72]
Cargoes	Secretory vesicles, Damaged mitochondria	mRNA, protein, lipid, etc.	[6, 73]
Major RNAs	mRNA	mRNA, miRNA, Long non-coding RNA (LncRNA)	[10, 74]
Biogenesis	Migration-dependent	Migration-independent, ECM-independent	[6, 73]
Location	At the junctions or tips of RFs	Multivesicular body	[6, 75]
Regulators	Sphingomyelin (SM), SMS2	Integrins, PI(4,5)P2, PIP5K, Rab35, Tetraspanins, cholesterol, Syt1, etc.	[10, 76]
Key Proteins	NDST1, PIGK, CPQ, EOGT, etc.	CD9, CD63, TSG101, Syntenin 1, etc.	[77–79]
Markers	Sphingomyelin, Rab35	ESCRTs, tetraspanins, ceramide, flotillins	[80, 81]
Involvement in migration	Yes, migration-associated	No, migration-independent	[24, 82]

Despite these promising findings, the study of migrasomes is still in its early stages, and many questions remain unanswered. The molecular mechanisms driving migrasome biogenesis, their full spectrum of biological functions, and their potential therapeutic applications require further investigation. In this review, we strive to elucidate the contemporary understanding of migrasomes, encompassing their genesis, structural characteristics, and functions in intercellular signaling. We will delve into their involvement in disease pathogenesis and examine their potential therapeutic applications, particularly in oncology, immunomodulation, and tissue regeneration. Furthermore, we will outline prospective research trajectories to fully harness their clinical potential.

## The biogenesis and molecular regulation of migrasome formation

**Fig. 2:**
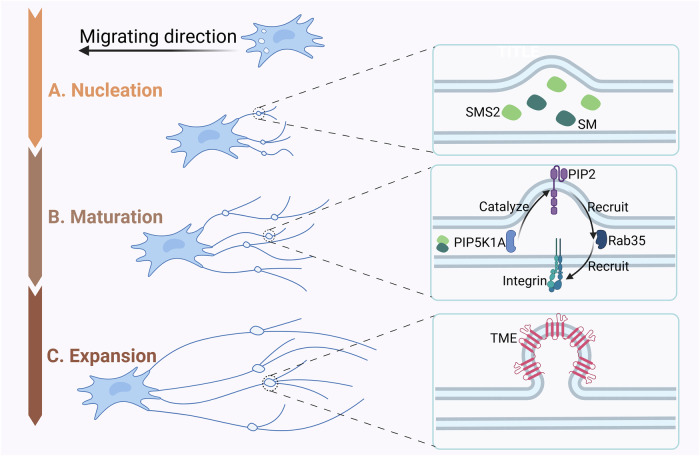
The formation of migrasomes. The biogenesis of migrating vesicles can be categorized into three distinct phases: **A** the nucleation phase, initiated by the focal assembly of sphingomyelin synthase 2 (SMS2); **B** the maturation phase, regulated by the phosphatidylinositol-4-phosphate 5-kinase (PIP5K1A)-Rab35-integrin signaling axis; and **C** the expansion phase, driven by the assembly of tetraspanin-enriched microdomains (TEMs) (Created with BioRender.com).

Migrasomes are dynamic, membrane-bound structures that form at the rear of migrating cells and play critical roles in cellular migration and interaction with the extracellular matrix (ECM). The formation of migrasomes involves a series of highly regulated molecular and biophysical processes, which can be divided into three key stages: nucleation, maturation, and expansion [16] (Fig. 2). Various factors, including signaling molecules, membrane lipids, and mechanical forces, are essential in regulating migrasome biogenesis, and these factors are closely linked to the migration patterns of cells.

### Nucleation: initial formation at RFs

Migrasomes primarily form at the ends or branching points of RFs, which are actin-rich structures located at the rear of migrating cells. The nucleation of migrasomes at these sites is a highly regulated process involving lipid composition and protein recruitment. A critical player in this process is sphingomyelin synthase 2 (SMS2), which catalyzes the conversion of ceramide into sphingomyelin (SM) in the plasma membrane [17]. SMS2 is recruited to the leading edge of the cell, where it forms stationary foci that eventually transition into migrasomes as the cell migrates. These SMS2 foci are essential for migrasome nucleation and represent the initial step in their formation. SM accumulation in these foci is crucial for the structural integrity of migrasomes [18]. Depletion of SM disrupts migrasome formation, underscoring its pivotal role in both initiating and maintaining the structural stability of migrasomes. Moreover, SM synthesis in migrasomes is dependent on SMS2 at migrasome formation sites (MFSs), which are located at the ends of RFs [19]. This de novo synthesis of SM further supports the formation and stabilization of migrasomes as the cell progresses along its migratory path.

Another key regulator of migrasome nucleation is phosphatidylinositol 4-phosphate 5-kinase (PIP5K1A), which converts PI4P into PI(4,5)P2. Recruitment of PIP5K1A to MFSs precedes the appearance of Rab35, a small GTPase that interacts with PI(4,5)P2. This interaction helps localize integrin α5 to MFSs, a crucial step for anchoring the RFs to the ECM; and facilitating cell migration [11, 20]. The early recruitment of these key molecular components establishes a platform for the further maturation and expansion of migrasomes.

### Maturation: integrins and tetraspanins at MFSs

Once migrasomes begin to form, a series of biochemical events drive their maturation. One of the first steps involves the recruitment of integrins to MFSs. Integrins, such as integrin α5, are enriched at MFSs before the appearance of TSPAN4, a tetraspanin protein that is a known marker for migrasomes [11]. These integrin-positive, TSPAN4-negative puncta at RFs serve as the primary anchoring points for RFs to the ECM, playing a critical role in the cell’s migration. The recruitment of Rab35 to MFSs by PI(4,5)P2 promotes the interaction between integrin α5 and the ECM, which is essential for securing RFs and supporting their retraction during cell movement [21].

Recent studies have also shown that the maturation of migrasomes involves the formation of tetraspanin-enriched microdomains (TEMs). Tetraspanins, such as TSPAN4, organize into these microdomains, which are essential for stabilizing and expanding migrasomes [12, 22]. Cholesterol is another critical factor in TEM formation and the stability of migrasomes. Depletion of cholesterol disrupts TEM formation, impairing both migrasome maturation and overall cell migration. The assembly of TEMs at MFSs into larger tetraspanin-enriched macrodomain (TEMA) structures further facilitates migrasome maturation, contributing to their structural integrity and function [22].

### Expansion: membrane tension and biophysical forces

After the nucleation and maturation phases, migrasomes proceed to the expansion phase, which is driven by a combination of biochemical interactions and mechanical forces. A key aspect of this process is tube pearling instability, a biophysical phenomenon where a cylindrical membrane structure undergoes periodic bulging or “pearling” due to mechanical stress [22]. This instability occurs when internal pressure and surface tension are not balanced, leading to localized swellings in the membrane. As RFs experience increased membrane tension, they become unstable, triggering the formation of these swellings that eventually develop into migrasomes.

Calcium plays a critical role in the expansion phase, particularly through the action of synaptotagmin-1 (Syt1), a calcium sensor protein. Syt1 is recruited to migrasomes and plays a vital role in stabilizing transient bulges formed due to tube pearling instability. The calcium-binding activity of Syt1 is crucial for the formation and stabilization of migrasome precursors, which are subsequently expanded through the action of tetraspanins. Tetraspanins, including TSPAN4, are incorporated into the migrasome membrane, stabilizing these structures and promoting their growth [23]. Tetraspanins also influence the mechanical properties of migrasomes. The formation of TEMAs increases the bending stiffness of the migrasome membrane, providing additional structural support. These TEMAs help stabilize the migrasomes by enhancing their membrane rigidity, which prevents them from collapsing under the mechanical stresses encountered during cell migration. The increased rigidity of TEMAs is largely due to their high cholesterol content, which strengthens the membrane by intercalating between phospholipid molecules [22]. The stabilizing role of tetraspanins is further demonstrated in recent studies using biomimetic systems. These studies show that, initially, migrasome precursors form as swellings on RFs devoid of TSPAN4. However, once TSPAN4 is incorporated, these swellings expand into fully formed migrasomes. This two-step process emphasizes the critical role of TSPAN4 in both the growth and stabilization of migrasomes [23].

### Regulation by migration dynamics

The formation and characteristics of migrasomes are significantly influenced by the dynamics of cellular migration. Studies have shown that cells exhibiting straight, persistent movement tend to generate more migrasomes, as the formation of RFs and migrasomes is closely linked to the linear, uninterrupted movement of the cell [24]. Faster, more directed cell movement results in longer RFs and more frequent migrasome formation at the RF ends. Conversely, cells with slower movement or more frequent changes in direction generate fewer migrasomes, likely due to the reduced stability and length of RFs. The impact of migration dynamics on migrasome formation is further emphasized by studies on vimentin, a cytoskeletal protein. Depletion of vimentin results in more frequent turning and slower migration, leading to fewer migrasomes. This highlights the critical role of migration persistence and speed in determining the number and size of migrasomes formed during cell migration [7].

## Methods for migrasome isolation, purification and characterization

### Isolation and purification of migrasomes

To understand migrasomes’ functions, isolating and purifying them from cellular or body fluid samples is essential. Several methods have been developed, notably from Yu’s lab [12], with others refining or proposing alternatives [25, 26]. Below, we outline the primary methods used for the isolation and purification of migrasomes, including both cell-derived and body fluid-derived migrasomes (Table 2).

**Table 2:** Current methods for migrasome isolation, purification and identification.

Step	Method	Description	Key Features/Notes
Extraction	Cell culture	Migrasomes are obtained from cell culture supernatants or attached to migrating cells.	Cells need to exhibit migration; RFs are critical.
	Mechanical collection	Migrasomes are harvested manually from migrating cell surfaces.	Time-consuming; requires cell migration to occur effectively.
	Microfluidics-based approaches	Uses microfluidic devices to guide and collect migrasomes from migrating cells.	Offers high precision; emerging technique.
Purification	Ultracentrifugation	Differential centrifugation to separate migrasomes from other vesicles.	Requires high-speed centrifugation; common method.
	Density Gradient centrifugation	Migrasomes are separated based on density using sucrose or iodixanol gradients.	High purity but labor-intensive; avoids contamination.
	Size Exclusion chromatography	Separates migrasomes based on size, removing smaller vesicles like exosomes.	Preserves structural integrity; suitable for downstream studies.
	Immunoprecipitation	Specific antibodies target migrasome surface markers (e.g., NDST1, PIGK).	Highly specific but costly; requires known markers.
	Filtration	Use of membrane filters with pore sizes >500 nm to isolate large migrasomes.	Simple but low specificity; suitable for initial separation.
Identification	Electron Microscopy (EM)	Transmission or scanning EM for detailed visualization of migrasome structure.	Gold standard; provides high-resolution images.
	Fluorescence microscopy	Migrasomes tagged with fluorescent markers for visualization and tracking.	Allows live imaging; requires fluorescent labeling.
	Western blotting	Detection of migrasome-specific proteins (e.g., NDST1, CPQ).	Semi-quantitative; requires validated markers.
	Mass spectrometry	Proteomics or lipidomics analysis to identify cargo and surface markers.	Comprehensive analysis of contents; high sensitivity.
	Flow cytometry	Size- and marker-based sorting using specific antibodies.	Enables high-throughput analysis; limited to known markers.
	RNA sequencing	High-throughput sequencing to analyze migrasome RNA cargo.	Reveals RNA profiles; requires purified migrasomes.
	Dynamic Light scattering (DLS)	Measures size distribution of migrasome populations.	Useful for assessing purity and size heterogeneity.

For cell-derived migrasomes, Yu’s sedimentation coefficient-based method involves multiple centrifugation steps and yields pure samples but requires specialized equipment [12]. Ma et al. suggested a size-based filtration technique suitable for high-throughput but may lose smaller migrasomes [27]. Lee’s team used an exosome isolation reagent for a cost-effective but less pure result [28]. Gu et al. enhanced Yu’s method with cytochalasin B and Ficoll gradient centrifugation, yielding high-quality samples but needing specialized reagents [12]. The method chosen depends on the required purity, yield, and speed, with Yu’s being resource-intensive and Ma’s offering a quicker option.

Isolating migrasomes from body fluids is more complex due to sample diversity. Yu’s adaptation of their cell-based method for serum involves extensive centrifugation and is effective but time-consuming [25]. Hu and Liu’s differential centrifugation for plasma and urine provides crude isolation but requires further purification [29]. Yang et al. developed a quicker method using WGA-coupled magnetic beads and flow cytometry for urine, showing promise for clinical applications like liquid biopsy [30].

### The characterization of migrasomes

The delineation of migrasome properties is pivotal for elucidating their physiological roles. To thoroughly investigate migrasomes, an integrative approach encompassing ultrastructural, proteomic, and genomics methodologies is utilized (Table 2).

#### Morphological evaluation

Morphological characterization is essential for distinguishing migrasomes from other extracellular vesicles. Migrasomes typically exhibit a spherical or oval shape with a diameter ranging from 0.5 to 3 μm, often displaying a distinctive pomegranate-like structure due to the presence of numerous smaller vesicles within them. Several advanced imaging techniques are used to analyze their morphology. Transmission electron microscopy (TEM) provides the highest resolution and is indispensable for visualizing the internal architecture of migrasomes, allowing researchers to observe their intricate substructure. However, this technique requires extensive sample preparation, including the creation of ultra-thin sections of the sample, which can be technically demanding [31]. Laser Scanning Confocal Microscopy (LSCM) although lower in resolution compared to TEM, is widely used for live-cell imaging and can capture dynamic changes in migrasome distribution. This method is especially valuable for studying the size and location of migrasomes in living cells, as it allows for real-time tracking of migrasome formation and movement without the need for extensive sample processing [16]. Live-cell Imaging is crucial for observing migrasome formation and dynamics in real-time, without compromising the integrity of the cells. It allows for the study of migrasome behavior under different physiological or experimental conditions, providing insights into their role in cellular processes like migration and communication [32]. Additionally, novel imaging technologies such as two-photon synthetic aperture microscopy, proposed by Dai et al., offer the advantage of reduced phototoxicity and provide multi-view, three-dimensional imaging in deep tissues, further enhancing the study of migrasomes in live organisms [26].

#### Protein marker detection

To identify and track migrasomes, the detection of specific protein markers is an essential component of their characterization. Several proteins are now recognized as migrasome markers, with TSPAN4 being one of the most prominent. Identified by Yu et al. through mass spectrometry, TSPAN4 is highly enriched in migrasomes and plays a crucial role in their biogenesis [12]. However, because TSPAN4 is also present in exosomes, its expression alone cannot definitively distinguish migrasomes from other vesicles. Therefore, a combination of protein markers and morphological analysis is often employed. Other markers, including NDST1, CPQ, PIGK, and EOGT, have been identified as relatively specific to migrasomes and can be used in conjunction with TSPAN4 to improve specificity. Notably, these markers are either absent or expressed at very low levels in exosomes, providing a means to differentiate migrasomes from other vesicles [33, 34]. Additionally, recent studies have suggested the potential of Rab10 and Caveolin-1 (CAV1) as markers for intraluminal vesicles within migrasomes, further refining the criteria for migrasome identification [35]. Fluorescent labeling techniques, such as TSPAN4-GFP fusion proteins or Wheat Germ Agglutinin (WGA)-based labeling, allow for real-time visualization of migrasomes in live cells. WGA specifically binds to sialic acid and N-acetylglucosamine on the surface of migrasomes, providing a non-invasive method to track these vesicles without disrupting their formation [36]. Additionally, nucleic acid dyes like SYTO14 have been used to stain RNA within migrasomes, although this method is less reliable due to variability in RNA content across different migrasomes [36].

#### Particle size analysis

Particle size analysis plays a critical role in distinguishing migrasomes from other types of extracellular vesicles. NTA is commonly employed to measure the size distribution of vesicles, including migrasomes. Studies have shown that migrasomes are typically larger than exosomes, with a reported diameter of 307.7 ± 6.0 nm—about 1.7 times larger than typical exosomes [27, 37]. This size difference provides a straightforward method for differentiating migrasomes from other vesicles. In addition to NTA, other methods such as dynamic light scattering and electron microscopy can provide complementary information on the size and morphology of migrasomes. The size distribution of migrasomes is also relevant for studying their biogenesis and release mechanisms, as well as their potential as therapeutic vehicles in drug delivery systems.

## The biological function of migrasomes

**Fig. 3:**
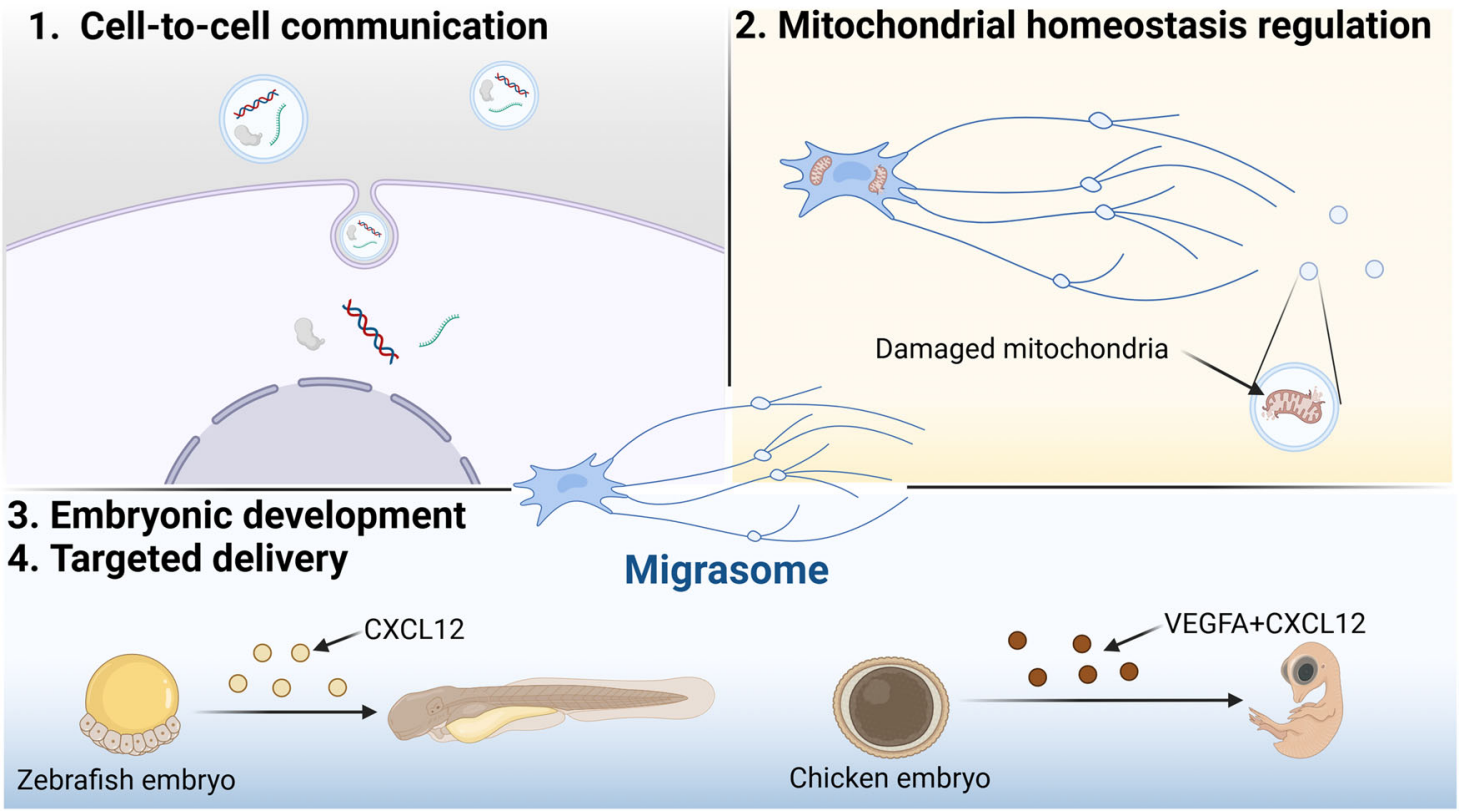
The biological function of migrasomes. Migrasomes play a pivotal role in various biological processes, including cell-to-cell communication, mitochondrial homeostasis regulation, embryonic development, and the targeted delivery of signaling molecules to spatially defined locations (Created with BioRender.com).

Recent researches showed that migrasome plays an important role in various fields including cell-to-cell communication, homeostasis maintenance, embryonic development, various diseases occurrence, progress and diagnosis. At present, the known functions of migrasomes are various, and we summarize them as follows (Fig. 3).

### Migrasomes and cell-to-cell communication

Migrasomes are rich repositories of cytokines, chemokines, growth factors, and diverse cellular constituents integral to signaling mechanisms. Upon the disintegration of migrasomes, the bioactive entities they harbor are disseminated, thereby enabling intercellular communication [38]. This process can elicit a wide range of responses, from promoting chemotaxis to influencing cell behavior during tissue development and immune responses. For example, in zebrafish embryonic development, migrating cells produce migrasomes enriched in CXCL12, which binds to its receptor CXCR4 on dorsal precursor cells (DFCs) [30]. This interaction guides the proper migration of DFCs, ensuring the correct localization and development of the organs. Similarly, migrating neutrophils leave behind prolonged trails of migrasomes rich in CXCL12, which serve as guidance cues for T cells to follow, thereby coordinating immune responses and cell migration patterns.

Migrasomes also serve as a unique conduit for lateral transfer of materials between cells. Recent studies have shown that migrasomes are not merely extracellular carriers but also actively mediate the transfer of mRNA, proteins, and other cellular components between cells. For instance, antigen-presenting dendritic cells (DCs) use migrasomes to transfer cytosolic components like chemokines and antigens to other DCs, influencing immune signaling and function [31]. Similarly, following traumatic brain injury (TBI), neutrophils utilize migrasomes to communicate with microglia, potentially modulating inflammation and repair processes [25]. The molecular cargo within migrasomes is diverse, encompassing proteins and mRNAs that play pivotal roles in cell function. For example, migrasomes have been shown to contain mRNA species related to cell metabolism, intracellular transport, and membrane structure. Notably, migrasomes can carry full-length genes like *Pten*, and transfer their mRNA to recipient cells. Migrasomes carrying *Pten* mRNA were co-cultured with PTEN-deficient tumor cells, leading to the restoration of PTEN expression and a subsequent reduction in AKT phosphorylation and cell proliferation [10]. This highlights the potential of migrasomes in correcting molecular defects in recipient cells, opening up possibilities for therapeutic applications. Additionally, the mechanism by which migrasomes are internalized by recipient cells, likely involving endocytosis or phagocytosis, remains a subject of active investigation. The ability of migrasomes to selectively concentrate specific mRNAs, like *Pten*, suggests a sorting mechanism that allows the transfer of functional genetic material between cells. This mechanism can bypass traditional pathways such as endo/lysosomal degradation, allowing mRNA to reach the cytosol and be translated into functional proteins that influence cellular behavior.

### Mitochondrial homeostasis regulation

Migrasomes play a pivotal role in maintaining cellular homeostasis, particularly in the regulation of mitochondrial health [39]. One of the most studied examples is the process of mitocytosis, a specialized quality-control mechanism for damaged mitochondria [40]. In cells undergoing migration, which experience heightened mitochondrial stress due to increased energy demands, damaged mitochondria are selectively transported to the cell’s periphery and sequestered into migrasomes [26]. This process occurs through the preferential interaction of damaged mitochondria with outward motor proteins, such as kinesin 1, while avoiding the inward motor proteins that drive mitochondrial movement towards the cell center. By encapsulating damaged mitochondria in migrasomes and expelling them from the cell, mitocytosis helps maintain mitochondrial integrity, preventing the accumulation of dysfunctional organelles that could compromise cellular function.

Mitocytosis is not exclusive to mitochondria; other organelles, such as autophagosomes, lysosomes, the endoplasmic reticulum, and lipid droplets, have also been found within migrasomes, suggesting that these vesicles could play a broader role in cellular homeostasis [41]. Although much is known about the mitochondrial quality control aspect, the role of migrasomes in managing other organelles and cellular components is still under investigation. For example, autophagosomes found in migrasomes have been shown to alleviate cellular stress, though the specific mechanisms of how these organelles contribute to homeostasis remain unclear.

Moreover, migrasomes are involved in maintaining the homeostasis of various physiological systems. In the cardiovascular system, TSPANs, key structural components of migrasomes, are involved in processes such as thrombosis, angiogenesis, and vascular injury, indicating that migrasomes play a role in vascular homeostasis [15]. In the central nervous system, migrasomes may affect the homeostasis of neural cells, with evidence suggesting that they participate in regulating the migration of neural crest cells (NCCs), which are critical for cranial development [42]. This link between migrasomes and the nervous system further underscores their importance in maintaining cellular and tissue integrity.

The selective cargo within migrasomes, particularly damaged mitochondria, raises intriguing questions about their broader role in cellular homeostasis. While mitocytosis serves to remove dysfunctional organelles, could migrasomes also facilitate the transfer of healthy organelles or other essential materials between cells? Are migrasomes acting as “supply stations” for essential molecules, or are they primarily “garbage cans” for waste products? These are critical questions that highlight the complexity of migrasome function and their potential contributions to both cellular maintenance and intercellular communication.

### Migrasomes and embryonic development

Migrasomes have emerged as essential players in the regulation of embryonic development, acting as dynamic carriers of signaling molecules that coordinate tissue morphogenesis, organ development, and cell migration. A key function of migrasomes in this context is their ability to transport and release a variety of bioactive factors, such as chemokines, growth factors, and morphogens, that influence the behavior of developing cells [12]. In particular, the chemokine CXCL12 has been identified as a crucial component of migrasomes that regulates the positioning and migration of precursor cells during organogenesis [13].

One of the most compelling studies demonstrating the role of migrasomes in embryonic development was conducted in zebrafish embryos. The study discovered that migrasomes, abundant in CXCL12, accumulate in the cavity beneath the embryonic shield during the gastrula stage [13]. These migrasomes act as signaling vehicles that interact with their receptor CXCR4 on dorsal forerunner cells (DFCs), guiding the migration and proper localization of these cells, which is essential for correct organ morphogenesis [30]. In a model where CXCL12a was knocked out, organ development was impaired, but exogenous administration of migrasomes from wild-type embryos was able to rescue this defect, underscoring the importance of migrasomes in embryonic signaling and tissue patterning.

In addition to their role in zebrafish, migrasomes have been shown to influence organ development in other species as well. For example, migrasomes in chick embryos, rich in pro-angiogenic factors such as VEGFA and CXCL12, have been found to promote angiogenesis and recruit monocytes to the developing chorioallantoic membrane [13]. This suggests that migrasomes are not only involved in cell migration but also in facilitating key processes like vascularization, which is critical for organ development. Similarly, in human mesenchymal stromal cells (MSCs), migrasomes enriched with stromal cell-derived factor 1 (SDF-1) have been shown to attract hematopoietic progenitors, indicating their role in bone marrow development and cell-to-cell communication during organogenesis [43].

The discovery of migrasomes involvement in the regulation of zebrafish gut development has provided further insights into their role in embryonic tissue formation. These migrasomes are particularly abundant in the mesoderm-endoderm syncytial layer, where they contribute to the proper localization of precursor cells and coordinate the asymmetric development of the gut. Notably, genetic knockout of TSPAN4a and TSPAN7, key components of migrasomes, leads to defective migrasome formation and resulting organ development defects [12]. This emphasizes that the integrity of migrasomes is crucial for the proper execution of embryonic development, as defective migrasome formation disrupts normal organogenesis.

The emerging role of migrasomes in embryonic development highlights their importance as mobile signaling hubs, capable of packaging and transporting critical molecular cues across developing tissues. Through their ability to mediate chemotaxis and direct cellular migration, migrasomes coordinate the precise spatial and temporal organization of cellular events necessary for proper organ formation. Further research into migrasomes’ diverse functions during development across different species will undoubtedly uncover additional mechanisms by which they regulate tissue morphogenesis, offering potential therapeutic insights for developmental disorders and diseases related to cell migration.

### Targeted delivery of signaling molecules to spatially defined locations

Migrasomes are abundant in cytokines, trophic factors, and morphogens, and are indispensable for orchestrating cell migration, organogenesis, and tissue morphogenesis by ensuring the spatial-temporal precision of signaling molecule localization [44]. The ability of migrasomes to selectively transport and release bioactive molecules at specific sites underscores their importance in regulating complex biological processes [45].

In the context of embryonic development, one of the most compelling examples of migrasomes’ targeted delivery function can be found in zebrafish embryos. During gastrulation, mesodermal and endodermal cells produce migrasomes that accumulate in specific regions beneath the embryonic shield, forming localized centers of signaling molecules like CXCL12, a key regulator of left-right asymmetry [12]. These migrasomes are crucial for the migration of DFCs, which cluster beneath the embryonic shield and contribute to the formation of Kupffer’s Vesicle (KV), a critical organ for establishing the body’s left-right symmetry [12]. In the absence of migrasomes, such as in Tspan4−/− zebrafish, DFC migration is disrupted, leading to impaired KV formation and abnormal organ positioning. This highlights how migrasomes can act as localized sources of signaling molecules, providing directional cues that guide cellular migration and organ development.

Further evidence of migrasomes’ role in targeted delivery is found in the chicken embryo, where monocyte-derived migrasomes are involved in angiogenesis [13]. As monocytes migrate along the CAM, they deposit migrasomes along their paths. These migrasomes, rich in pro-angiogenic factors such as VEGFA and CXCL12, help create a favorable microenvironment for capillary formation, promoting the development of vascular networks. Unlike in zebrafish, where migrasomes detach and diffuse through the embryonic cavity, in the chicken embryo, migrasomes adhere to the extracellular matrix, forming linear tracks that correspond to the migratory pathways of monocytes. This demonstrates how migrasomes can generate spatially organized gradients of signaling molecules, influencing the formation of structured tissue patterns like capillary networks.

Recent studies have also highlighted the role of migrasomes in immune system regulation. Migrasomes produced by MSCs contain signaling molecules like CXCL12, which acts as a chemoattractant for hematopoietic progenitor cells and leukemia cells [46, 47]. This suggests that migrasomes may play a crucial role in guiding cell migration during immune responses, as well as in tissue regeneration and repair.

The targeted delivery of signaling molecules via migrasomes not only provides a mechanism for precise spatial and temporal regulation of development but also opens up new avenues for understanding complex biological processes. These discoveries suggest that migrasomes may be central to many physiological and pathological events, including embryonic development, immune response, angiogenesis, and potentially even cancer metastasis. As research continues, the potential therapeutic applications of migrasomes—such as targeted drug delivery or tissue engineering—are becoming increasingly evident, offering exciting possibilities for advancing regenerative medicine and treating diseases associated with cell migration and signaling defects.

## The biological role of migrasomes in diseases

**Fig. 4:**
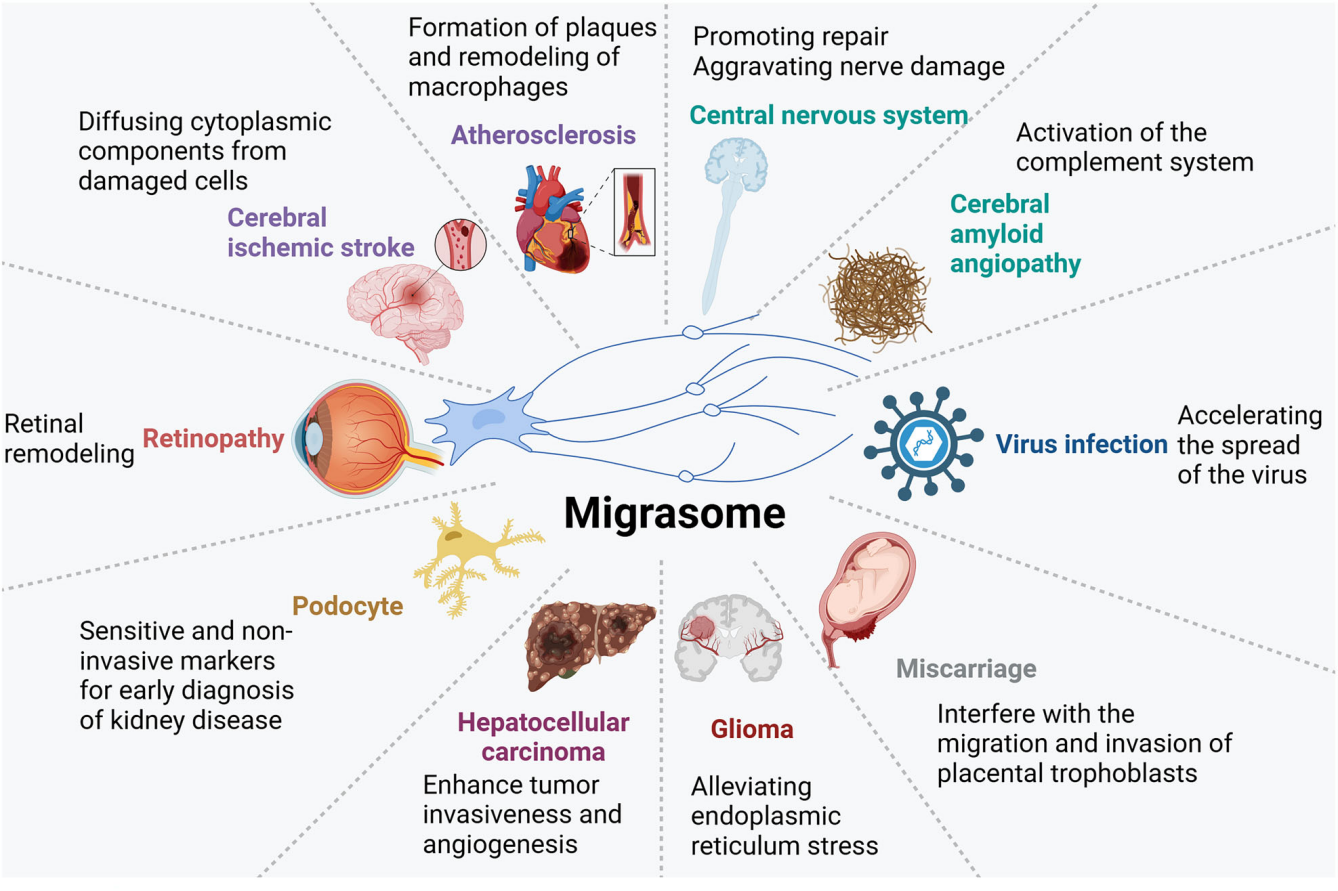
The biological role of migrasomes in diseases. Migrasomes are implicated in the pathogenesis of both non-neoplastic diseases and malignant diseases, highlighting their potential role in disease progression and therapeutic intervention (Created with BioRender.com).

In contrast to the previously mentioned physiological roles of migrasomes, the potential clinical use of migrasome modulation remains a relatively uncharted territory, despite ongoing research into the association between migrasomes and various diseases. To date, several studies have highlighted links between migrasomes and a range of health conditions (Fig. 4).

### Nonneoplastic diseases

#### Cardiovascular diseases

Migrasomes, membrane-bound vesicles involved in cell migration, have recently emerged as important players in cardiovascular diseases, with increasing evidence linking them to atherosclerosis, myocardial infarction (MI), and ischemic injury. These vesicles, which are rich in signaling molecules and bioactive compounds, play a crucial role in mediating cellular communication and influencing the local microenvironment, thus impacting the progression of cardiovascular conditions.

One of the key mechanisms through which migrasomes influence cardiovascular health is their involvement in atherosclerosis. Recent studies have highlighted the association between migrasomes and macrophage activity in the context of atherosclerotic plaque dynamics. For instance, TSPAN4, a tetraspanin protein essential for migrasome formation, has been shown to be highly expressed in macrophages associated with atherosclerosis regression, intraplaque hemorrhage, and plaque rupture [48]. In mouse models, TSPAN4 expression is upregulated in response to spontaneous and induced MI, suggesting that migrasomes may play a role in modulating the macrophage response during atherosclerosis progression [48]. Migrasomes may thus serve as a novel therapeutic target to influence macrophage behavior and promote plaque stability or regression, offering potential avenues for treating atherosclerosis and reducing the risk of cardiovascular events such as stroke or MI.

In the context of ischemic injury, migrasomes have been implicated in conditions like ischemic stroke, which results from the blockage of blood flow to the brain. It has been shown that a high-salt diet exacerbates the outcomes of acute ischemic stroke by increasing migrasome formation in neurons surrounding the infarcted area [15, 40]. These migrasomes appear to disperse cytosolic components from damaged cells, potentially contributing to the pathological cascade of ischemic injury. Notably, the presence of migrasomes has been detected in the infarcted brain parenchyma of stroke patients, further suggesting their involvement in the ischemic process. The role of migrasomes in ischemic stroke opens up new possibilities for targeted therapies aimed at regulating their formation or function to mitigate brain damage and improve recovery following stroke.

Beyond ischemic conditions, migrasomes may also be involved in vascular homeostasis and the repair of damaged blood vessels. Migrasomes have been shown to participate in endothelial and smooth muscle cell migration, which is critical for wound healing and tissue regeneration following vascular injury [49]. Their ability to transport key signaling molecules could influence the formation of new blood vessels (angiogenesis) and the stabilization of vascular structures, particularly in the aftermath of atherosclerotic plaque rupture or myocardial infarction.

Despite the emerging evidence linking migrasomes to cardiovascular diseases, much remains to be understood about their precise mechanisms of action. Research is ongoing to explore how migrasomes influence inflammation, endothelial function, and the remodeling of vascular tissues. With further studies, migrasomes may prove to be valuable biomarkers for early detection of cardiovascular diseases, as well as therapeutic targets to modulate disease progression and improve outcomes for patients suffering from atherosclerosis, myocardial infarction, and stroke.

#### Urinary diseases

Migrasomes, specialized vesicles involved in cell migration, are gaining recognition for their significant roles in the development and progression of urinary diseases, particularly those affecting the kidneys. Emerging research has identified migrasomes as sensitive and reliable biomarkers for early detection of podocyte injury, a key factor in various kidney diseases. Podocytes are specialized cells in the glomerulus that play a crucial role in maintaining the filtration barrier of the kidney, and their damage is often associated with proteinuria, a hallmark of many renal conditions.

One of the most striking findings is that migrasomes are secreted in large quantities during podocyte injury, often before the appearance of proteinuria, which has traditionally been used as a diagnostic marker for kidney damage. For instance, In both human and murine models of podocyte injury induced by puromycin aminonucleoside (PAN), the secretion of migrasomes was significantly increased [36]. These migrasomes were detected in the urine before the onset of proteinuria, suggesting that they could serve as an earlier and more sensitive indicator of renal damage. Similarly, increased numbers of migrasomes have been found in the urine of diabetic nephropathy patients, even when proteinuria levels remained below 5.5 g/day, further supporting their potential as biomarkers for early-stage kidney disease [50].

In addition to podocyte injury, migrasomes are also implicated in the pathophysiology of other forms of renal damage. Studies have shown elevated levels of migrasomes in the urine of patients with various kidney diseases, including those with glomerulonephritis and diabetic nephropathy. These findings suggest that migrasomes may reflect not only podocyte injury but also broader glomerular and renal damage. As such, urinary migrasomes hold promise as a novel, non-invasive marker for the early diagnosis and monitoring of kidney diseases, potentially allowing for earlier intervention and better management of renal conditions.

#### Neurological diseases

Migrasomes, specialized vesicles formed during cell migration, have increasingly been implicated in various neurological diseases, particularly in the context of neurodegeneration, ischemic injury, and the disruption of the blood-brain barrier (BBB). Recent research has revealed that migrasomes play crucial roles in the clearance of damaged neuronal material, modulation of inflammation, and the progression of several neurological conditions, providing new insights into the mechanisms underlying brain pathologies [51].

One significant finding is the role of migrasomes in the clearance of neuronal debris following neuronal damage. In studies examining ischemic injury to the central nervous system (CNS), migrasomes were found to accumulate near atrophic neurons, often enriched with neuronal fragments [29]. This suggests that migrasomes may mediate the removal of damaged neuronal components, potentially contributing to neuronal atrophy or exacerbating the injury. In vitro experiments also demonstrated that a high-salt environment can promote migrasome formation by microglia, cells that play a pivotal role in CNS immune responses. Interestingly, high salt not only increased migrasome production but also enhanced the pro-inflammatory polarization of microglia, suggesting a complex role for migrasomes in both cellular repair and inflammation. Whether this migrasome-mediated clearance process involves alternative pathways to apoptosis, and how it influences the progression of neurological diseases, remains an area for further exploration.

Migrasomes have also been implicated in cerebral amyloid angiopathy (CAA), a condition characterized by the deposition of amyloid beta-proteins (Aβ) in cerebral blood vessels, leading to blood vessel degeneration, hemorrhages, and cognitive decline [51]. In CAA, the overactivation of the complement system plays a critical role in exacerbating brain dysfunction and BBB damage. Migrasomes derived from macrophage-lineage cells were found to carry complement activation-related molecules, including CD5L, which further promote complement-dependent BBB disruption. These findings suggest that migrasomes and their cargo may be central in the pathophysiology of CAA, with CD5L and migrasomes potentially serving as therapeutic targets to modulate complement activation and slow disease progression.

In addition to CAA, migrasomes have been found to contribute to ischemic brain injury. A study showed that high-salt diets were shown to exacerbate ischemic damage in the brain, promoting the formation of migrasomes rich in neuronal debris in the infarcted brain tissue [40, 49]. These migrasomes were frequently located near damaged or atrophic neurons, suggesting their involvement in either the clearance of cellular debris or further contributing to neuronal damage. This dual role underscores the need for further research into the precise functions of migrasomes in ischemic conditions, particularly in terms of their potential to influence neuronal survival and recovery.

#### Virus infection

Migrasomes have been increasingly recognized for their involvement in virus infections. Emerging research indicates that migrasomes may play significant roles in viral transmission, immune response modulation, and the spread of infectious agents within the host. Their ability to transport viral particles and interact with host cells suggests they could be key players in the pathogenesis of viral diseases.

One of the most notable discoveries in this area is the observation that migrasomes are involved in the spread of poxviruses, such as vaccinia virus (VACV) [52]. During the late stages of vaccinia virus infection, migrasomes were formed, containing intracellular mature virions (IMV) and extracellular enveloped virions (EEV), both of which are important for viral transmission. This suggests that migrasomes might facilitate the movement of viral particles between infected and uninfected cells, contributing to the spread of the virus. Furthermore, their research indicated that external factors, such as nanoplastics, can enhance the ability of poxviruses to be transmitted via migrasomes, highlighting a potential environmental modulator of viral transmission.

In a similar vein, herpes simplex virus type 2 (HSV-2) has also been shown to utilize migrasomes for transmission [53]. A study demonstrated that HSV-2-infected cells release migrasomes that contain viral particles, and these migrasomes can then transfer HSV-2 to uninfected cells, resulting in productive infection. This finding underscores the role of migrasomes in viral dissemination, particularly in the early stages of infection, where the migration of viral material can lead to rapid propagation of the virus.

Migrasomes also play a role in the immune response during viral infections. For example, platelets infected with SARS-CoV-2 were found to internalize the virus, and the subsequent programmed cell death of these platelets led to the release of migrasomes containing thrombotic and pro-inflammatory contents [54]. These migrasomes can potentially contribute to thrombo inflammation, a key feature of COVID-19, by triggering immune activation and exacerbating vascular damage. This raises the possibility that migrasomes might not only serve as carriers for viral particles but also influence host immune responses, promoting inflammation or immune dysregulation in viral infections.

The potential of migrasomes to serve as vectors for viral transmission and immune modulation extends to other viral infections, such as vaccinia virus and herpes simplex virus [52]. Studies have shown that migrasomes, formed after infection, can carry viral proteins (such as MPXVgp045 from poxviruses) and facilitate their spread through infected tissues. These findings suggest that migrasomes may be an important route for viruses to invade new cells and tissues, potentially bypassing the usual immune defenses.

Additionally, the possibility that migrasomes could be harnessed for vaccine delivery has been raised. Given their ability to transport viral particles and modulate immune responses, migrasomes could potentially be engineered to carry antigens or other therapeutic molecules to target cells, offering a novel approach to infection prevention and immune system activation.

#### Retinopathy

Migrasomes, a newly recognized type of EVs, are emerging as key players in the pathogenesis of retinopathy, particularly in conditions such as proliferative vitreoretinopathy (PVR) [55, 56]. PVR is a severe complication that often arises following retinal detachment or retinal injury, characterized by the formation of a fibrovascular membrane that can lead to vision impairment or blindness. Recent studies have highlighted the significant roles that migrasomes play in the progression of PVR, particularly in the migration and proliferation of retinal pigment epithelial (RPE) cells, which are central to the disease’s development. One of the pivotal findings in this area is the work by Wu et al., who identified TSPAN4, a migrasome marker, as being abundantly expressed in clinical samples related to PVR [55]. TSPAN4 is associated with migrasome formation, and its presence in the RPE cells suggests that migrasomes may mediate key cellular processes in the pathogenesis of PVR. Specifically, the internalization of migrasomes by RPE cells was found to enhance their migration and proliferation capabilities, both of which are critical for the formation and contraction of the PVR membrane. These findings suggest that migrasomes contribute to the pathological remodeling of the retinal tissue, driving the progression of the disease. In addition to the role of migrasomes in RPE cells, it remains unclear whether migrasomes produced by RPE cells themselves could further influence the development of PVR. Since migrasomes are known to facilitate cellular communication and material transport, it is plausible that they might act as signaling mediators between different retinal cell types, promoting the recruitment of inflammatory or proliferative cells that contribute to PVR pathology. This raises the question of whether targeting migrasome formation or its associated pathways could provide a therapeutic strategy to halt or slow the progression of PVR.

#### Other disease

Migrasomes have been implicated in various disease processes, highlighting their potential role in conditions ranging from reproductive health to chronic inflammatory diseases. These vesicles are increasingly recognized for their involvement in cell migration, immune regulation, and tissue remodeling, making them significant in the pathogenesis of diseases like abortion, inflammatory bowel disease (IBD), and other chronic conditions [57].

In the context of abortion, recent research has shown that nanoplastic pollution can negatively impact women’s reproductive health. Wan et al. demonstrated that exposure to polystyrene nanoplastics (PS-NPs) led to miscarriages in pregnant mice [37]. PS-NP exposure inhibited ROCK1-mediated cell migration, invasion, and migrasome formation in trophoblast cells, crucial for normal implantation and pregnancy. Mechanistically, PS-NPs induced autophagy, leading to the degradation of SOX2, a transcription factor necessary for the expression of ROCK1. The reduced transcription of ROCK1 impaired trophoblast cell functions, resulting in miscarriage. Restoring SOX2 or ROCK1 levels mitigated these effects and reduced miscarriage rates, suggesting that migrasomes could play a key role in maintaining pregnancy. This finding points to migrasomes as critical mediators of reproductive health, and further research into their precise physiological roles could open new avenues for addressing pregnancy complications.

Similarly, as a chronic condition marked by nonspecific inflammation of the intestinal mucosa, IBD may also involve migrasomes in its pathogenesis [57]. Migrasomes have been shown to influence immune responses and the integrity of the intestinal mucosal barrier. IBD is associated with barrier dysfunction, which exacerbates chronic inflammation. Previous studies on other EVs, such as exosomes, have demonstrated their dual role in IBD-either promoting inflammation or aiding tissue repair. For instance, exosomes can impair the intestinal barrier and promote M1 macrophage polarization through the delivery of inflammatory microRNAs, contributing to disease progression. However, they can also activate repair mechanisms, such as promoting the repair of epithelial cells through signaling molecules like ANXA1 and miR-195a-3p [58–60]. As migrasomes are similar to exosomes in their vesicular nature, they may exert similar, yet distinct, effects at different stages of IBD. By participating in either pro-inflammatory or reparative processes, migrasomes could influence the outcome of IBD, offering new possibilities for therapeutic interventions, including the modulation of EV-based therapies currently under clinical investigation.

### Malignant diseases

Migrasomes are emerging as crucial players in the progression and metastasis of various malignancies. Their roles in cancer biology are multifaceted, influencing tumor cell viability, invasion, angiogenesis, and immune evasion. Recent studies have provided compelling evidence for the significant impact of migrasomes in malignant diseases such as glioma and hepatocellular carcinoma (HCC), among others.

In the case of glioma, proteomic analyses have revealed that migrasomes derived from glioma cells are enriched with LC3B-positive autophagosomes. The inhibition of autophagosome-lysosome fusion increases the number of autophagosomes, subsequently promoting migrasome formation and alleviating endoplasmic reticulum (ER) stress [28]. This process highlights the critical role of migrasomes in maintaining glioma cell viability under stress conditions. Furthermore, bioinformatic analyses by Zheng Y et al. have identified TSPAN4 as a key player in the progression of glioblastoma multiforme (GBM) and lower-grade gliomas (GBMLGG), suggesting that migrasomes may exacerbate the malignant progression of these tumors [28, 61, 62]. However, the specific roles and mechanisms of TSPAN4 in this context require further elucidation to confirm its potential as a diagnostic, prognostic, and therapeutic target.

In HCC, TSPANs, particularly CD151, have been found to be aberrantly increased in HCC tissues [63]. This increase is strongly associated with TSPAN4 levels and predicts poor prognosis. Mechanistic studies have shown that the abnormal elevation of CD151 promotes migrasome formation, enhancing the invasiveness and angiogenesis of HCC cells. Targeting the migrasome formation driven by elevated CD151 levels presents a promising anti-angiogenic therapeutic strategy for HCC, offering new insights into the mechanisms behind HCC metastasis.

Although current research on migrasomes in hematopoietic malignancies and solid tumors remains limited, pan-cancer analyses using bioinformatic methods have revealed that migrasome-related genes are frequently upregulated in various tumors and are closely associated with poor prognosis. These studies have shown that migrasome scores correlate with tumor immunity, stroma scores, macrophage abundance, immune checkpoint genes, tumor mutational burden (TMB), and microsatellite instability (MSI). For instance, Qin et al. identified migrasome-related genes as potential immunotherapeutic targets, where high expression levels predict poor prognosis [64]. Additionally, blocking the intrinsic migration-promoting effect of migrasomes using nanoparticles has been proposed as a novel strategy for anti-metastasis nanotherapy, offering new insights into the role of migrasomes in tumor prognosis and immunotherapy.

## Potential therapeutic applications of migrasomes

Migrasomes have attracted significant attention due to their potential roles in disease mechanisms and therapeutic applications. These lipid bilayer-bound structures are formed during cellular migration and are involved in various cellular functions such as signal transduction, tissue development, immune regulation, and intercellular communication [35, 44, 51]. Their ability to transport biomolecules makes them promising candidates for therapeutic targets in diseases.

In cardiovascular diseases, migrasomes play a key role in tissue repair and regeneration, especially following ischemic injuries such as heart attacks or strokes [40, 48, 65]. Secreted by migrating cardiac or endothelial cells, they promote cell proliferation, migration, and new blood vessel formation, aiding recovery. They also influence inflammatory responses, which may enhance healing and reduce fibrosis [57, 66, 67]. In the nervous system, migrasomes are involved in brain response after injuries like ischemic stroke or TBI [29, 68]. They contribute to tissue remodeling, the formation of new neurons, and the repair of the blood-brain barrier. In neurodegenerative diseases like Alzheimer’s and Parkinson’s, migrasomes might help clear toxic protein aggregates, potentially slowing disease progression. In renal health, particularly in glomerular diseases such as diabetic nephropathy or podocyte injury, migrasomes released by injured kidney cells could signal and mediate repair processes. Their ability to transport signaling molecules makes them potential biomarkers for assessing kidney function and disease progression. Continued research on migrasomes could lead to innovative therapeutic strategies, utilizing their natural roles in disease modulation and tissue regeneration. In cancer, migrasomes play a crucial role in the progression of gliomas, HCC, and pancreatic cancer, especially in metastasis [35, 63, 69, 70]. They enhance tumor cell movement and invasiveness, promote the formation of new blood vessels, and support the survival of circulating tumor cells, all essential for cancer spread. Furthermore, migrasomes help tumors evade immune detection, suggesting that inhibiting them could improve the effectiveness of immunotherapy. For instance, targeting tetraspanin proteins like CD151, which are essential for migrasome formation [63], might reduce cancer cell movement and their ability to metastasize. Additionally, migrasomes hold potential as cancer biomarkers, detectable in blood or other body fluids, offering a non-invasive way to monitor metastatic progression and response to treatment.

In summary, migrasomes represent a versatile and promising therapeutic target across various diseases. Their ability to mediate cell communication, enhance tissue repair, and modulate immune responses provides opportunities for novel treatments in cancer, cardiovascular disease, neurological disorders, and renal diseases. Further research into the mechanisms by which migrasomes function and their potential therapeutic uses could lead to the development of innovative treatments that capitalize on their natural roles in disease modulation and tissue regeneration.

## Future perspectives of migrasome

Migrasomes have rapidly gained attention within the scientific community due to their potential roles in cellular communication and disease pathogenesis [39, 71]. Although the study of migrasomes is still in its early stages, mounting evidence suggests that these organelles play crucial roles in a wide array of physiological and pathological processes, such as embryonic development, wound healing, and cancer metastasis. Migrasomes are found across various cell types, tissues, and species, further underscoring their biological significance. Their biogenesis involves a multi-step process, including nucleation, maturation, and expansion, with TSPANs (tetraspanins) emerging as key regulatory proteins that orchestrate their formation and cargo sorting [15, 38]. Functionally, migrasomes are analogous to exosomes but are unique in their specific association with migrating cells. Migrasomes facilitate the preservation of homeostasis via migracytosis, a mechanism in which migrating cells release these vesicles during their locomotion. The composition and molecular cargo of migrasomes are thought to reflect the physiological state of the originating cells, which may have implications for disease diagnosis and therapeutic interventions. Notably, recent studies have highlighted the importance of migrasomes in tumor progression and metastasis, where they may participate in modulating the tumor microenvironment and promoting cancer cell dissemination.

However, significant challenges remain in fully understanding the biogenesis and biological roles of migrasomes. One major obstacle is the lack of standardized methods for isolating and purifying migrasomes. Current protocols, while effective, are labor-intensive, require large sample volumes, and are time-consuming, making high-throughput studies difficult. In particular, the need for efficient and scalable purification techniques remains a major bottleneck in both basic research and clinical applications. Recent advances in microfluidic technologies and automated systems could offer promising solutions to these challenges by enabling the isolation of migrasomes from smaller sample volumes and providing higher throughput with improved purity (e.g., using nanoparticle-based capture methods or high-resolution imaging techniques to selectively isolate migrasomes from cell culture supernatants). Establishing robust and reproducible protocols for migrasome isolation and detection would also facilitate their application in diagnostics and therapeutics, akin to the standardized protocols used for other extracellular vesicles.

Moreover, while migrasomes are known to be produced on RFs and have a limited lifespan, the precise timing of their formation and release remains unclear. A better understanding of the dynamic changes in migrasome numbers in response to both normal and pathological cell migration is essential for deciphering their biological significance. Emerging research has suggested that migrasome quantity may correlate with the mobility of the cells they originate from, but this relationship is likely more complex than previously thought. It is important to consider not only the quantity but also the quality of migrasomes, as their cargo composition might vary in different contexts, such as inflammation, cancer, or neurodegeneration. Therefore, further studies are needed to explore the heterogeneity of migrasomes in terms of their molecular content and how these variations impact cellular interactions and disease progression.

One promising area for future research is the investigation of the molecular mechanisms underlying cargo sorting within migrasomes. Understanding how specific proteins, lipids, and RNAs are selectively packaged into migrasomes will be critical for elucidating their roles in both health and disease. Recent advances in single-cell RNA sequencing and proteomics have enabled more detailed profiling of extracellular vesicles, including migrasomes, and these techniques hold great potential for uncovering the specific cargo signatures associated with various pathologies. Moreover, elucidating the molecular machinery responsible for cargo sorting and vesicle trafficking will provide insights into the regulation of migrasome formation and may reveal new therapeutic targets for modulating migrasome-mediated pathways.

Another promising research direction involves the development of lineage-specific migrasome labeling techniques. Current methods for tracking migrasomes in vivo are limited, and novel labeling strategies are necessary to trace the origin and fate of these organelles more accurately. Advances in genetically encoded fluorescent markers and nanoparticle-based tracers could enable real-time visualization of migrasomes in living organisms, providing a powerful tool for understanding their dynamics during cellular migration and disease progression. Furthermore, lineage-specific labeling could help determine the impact of migrasomes on neighboring cells and tissues, shedding light on their potential roles in intercellular communication, immune modulation, and tissue remodeling.

In summary, while our understanding of migrasomes is still in its infancy, these emerging organelles hold great promise for advancing our knowledge of cellular migration, intercellular communication, and disease mechanisms. As research progresses, it is likely that migrasomes will become key players in diagnostic and therapeutic strategies, particularly in the fields of cancer, inflammation, and regenerative medicine. However, the development of standardized methodologies for migrasome isolation, cargo analysis, and lineage tracing will be crucial for unlocking their full potential in both basic research and clinical applications.
